# Cranberry proanthocyanidins inhibit esophageal adenocarcinoma *in vitro* and *in vivo* through pleiotropic cell death induction and PI3K/AKT/mTOR inactivation

**DOI:** 10.18632/oncotarget.5586

**Published:** 2015-09-10

**Authors:** Laura A. Kresty, Katherine M. Weh, Bree Zeyzus-Johns, Laura N. Perez, Amy B. Howell

**Affiliations:** ^1^ Division of Hematology and Oncology, Department of Medicine, Medical College of Wisconsin, Milwaukee, Wisconsin, USA; ^2^ Marucci Center for Blueberry and Cranberry Research, Rutgers University, Chatsworth, New Jersey, USA

**Keywords:** autophagy, cranberry proanthocyanidins, esophageal adenocarcinoma, bile acid, PI3K/AKT/mTOR

## Abstract

Cranberries are rich in bioactive constituents known to improve urinary tract health and more recent evidence supports cranberries possess cancer inhibitory properties. However, mechanisms of cancer inhibition by cranberries remain to be elucidated, particularly *in vivo*. Properties of a purified cranberry-derived proanthocyanidin extract (C-PAC) were investigated utilizing acid-sensitive and acid-resistant human esophageal adenocarcinoma (EAC) cell lines and esophageal tumor xenografts in athymic NU/NU mice. C-PAC induced caspase-independent cell death mainly via autophagy and low levels of apoptosis in acid-sensitive JHAD1 and OE33 cells, but resulted in cellular necrosis in acid-resistant OE19 cells. Similarly, C-PAC induced necrosis in JHAD1 cells pushed to acid-resistance via repeated exposures to an acidified bile cocktail. C-PAC associated cell death involved PI3K/AKT/mTOR inactivation, pro-apoptotic protein induction (BAX, BAK1, deamidated BCL-xL, Cytochrome C, PARP), modulation of MAPKs (P-P38/P-JNK) and G_2-_M cell cycle arrest *in vitro*. Importantly, oral delivery of C-PAC significantly inhibited OE19 tumor xenograft growth via modulation of AKT/mTOR/MAPK signaling and induction of the autophagic form of LC3B supporting *in vivo* efficacy against EAC for the first time. C-PAC is a potent inducer of EAC cell death and is efficacious *in vivo* at non-toxic behaviorally achievable concentrations, holding promise for preventive or therapeutic interventions in cohorts at increased risk for EAC, a rapidly rising and extremely deadly malignancy.

## INTRODUCTION

Esophageal adenocarcinoma (EAC) rates have increased over 500% during the last thirty years identifying EAC as the major histologic subtype of esophageal cancer and the fastest increasing of all cancer types in the US [1, 2]. In 2015, 16,980 new incident cases and 15,590 deaths due to esophageal cancer are estimated, representing the 7^th^ leading cause of cancer related deaths among US males [3]. Mortality and incidence statistics closely parallel one another reflecting poor survival due to ineffective options for screening, prevention and treatment coupled with late stage diagnosis. The precise reasons for the rapid increase in EAC and the only known precursor lesion, Barrett's esophagus (BE), are still being unraveled. However, persistent, symptomatic, reflux of gastric and duodenal contents, known as gastroesophageal reflux disease (GERD), have long been known to correlate with the development of BE and EAC [4, 5]. Heartburn is the primary symptom of GERD and is estimated to impact 60 million Americans [4]. In addition, obesity is reported to impart a 2 to 2.5-fold increase risk for EAC and a 1.5 to 2-fold increase risk for GERD [5]. Thus, there is a large population at elevated risk for BE and EAC, illustrating the potential global health significance of this growing problem. Other risk factors linked to the development of EAC include specific nutritional factors, overall dietary patterns, animal-based diets and the presence of hiatal hernia [6-8]. Plant-based diets rich in fruits, vegetables and fiber have generally been associated with EAC risk reduction [6] leading our laboratory to evaluate well characterized purified plant-derived extracts as potential inhibitors.

The urinary tract health benefits of cranberries are attributable to the unique A-type linkages in cranberry proanthocyanidins (C-PACs) which prevent adhesion of p-fimbriated uropathogenic E. coli [9–14]. More recently, cranberries have been reported to possess cancer inhibitory properties based on a large number of *in vitro* studies utilizing diverse cell lines of prostate, breast, cervical, ovarian, oral, stomach, bladder, neuroblastoma, lung and esophageal origin [15–23]. However, there is limited mechanistic information regarding the ability of cranberry constituents to inhibit cancers, especially *in vivo*. A small number of xenograft studies delivering cranberry extracts by intraperitoneal injections have shown positive results, supporting that direct administration increases tumor latency and inhibits growth of gastric, colon, prostate, lymphoma and glioblastoma cancer cells [15, 21–23]. To our knowledge only two *in vivo* studies report cancer-linked inhibitory effects following oral delivery of cranberry products. Boateng *et al*. indicated that 20% cranberry juice reduces azoxymethane-induced aberrant crypt foci [24]; whereas, Prasain and colleagues reported that cranberry juice inhibits nitrosamine-induced urinary bladder cancer development by 38% [25], but lacked any mechanistic explanation.

In this series of studies, we sought to investigate mechanisms associated with the cancer inhibitory potential of C-PAC, a well characterized proanthocyanidin-rich cranberry extract, utilizing a panel of authenticated EAC cell lines and conducting parallel molecular evaluations in the first *in vivo* study focused on C-PAC inhibition of EAC. Clinical and preclinical research efforts support that alterations in the susceptibility to cell death underlie neoplastic progression of Barrett's to EAC. In addition, acid refluxant is linked to alterations of inflammatory molecules, NF-kB signaling, PI3K/AKT/mTOR activation and MAPK signaling, ultimately resulting in an apoptosis resistant phenotype [26–31]. Targeting these pathways is logical for the prevention of esophageal cancer and potentially other cancers in which inflammation and aberrant cell death pathways provide a growth advantage and support resistance to treatment.

## RESULTS

### C-PAC induced G2-M cell cycle arrest and cell line specific S-phase delay accompanied by morphological changes consistent with cell death induction

**Figure 1:**
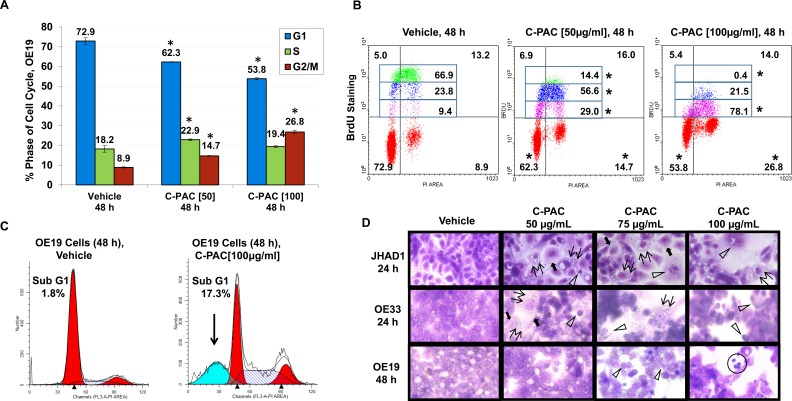
Effect of C-PAC on cell cycle distribution of EAC cells EAC cells were treated with C-PAC [50 or 100 μg/ml] for 24 and 48 hours, stained with PI alone or PI in combination with BrdU to determine cell-cycle phase and evaluate S-phase distribution. Cells were analyzed in triplicate for each condition with representative data shown as mean percentages + SEM. *P < 0.05 indicates a significant difference between C-PAC and vehicle treated cells, two-tailed Students *t*-test. **A.** C-PAC treatment significantly altered phase of cell cycle distribution in OE19, JHAD1 and OE33 cells (Supplemental Figure 1S, A and B). **B.** C-PAC induced a significant and dose-dependent S-phase delay in OE19 cells based on combined PI/BrdU staining. The upper left and upper right quadrants of each treatment panel represent early and late S-phase, respectively. The S-phase fraction was further quantitated for the intensity of BrdU incorporation by tertiles to examine differences in proliferative potential. C-PAC reduced BrdU incorporation as indicated by compression of the Y-axis intensity values (*P < 0.05). C-PAC treatment of OE33 cells did not result in an S-phase delay (Supplemental Figure 1S, A-C). **C.** C-PAC treatment decreased cells in G_1_, increased cells in G_2_ and caused a significant increase on the sub-G_1_ peak indicative of late apoptosis. **D.** C-PAC treatment altered EAC cellular morphology. Double arrows indicate formation of vacuoles and solid arrows show cytoplasmic swelling with intact membranes associated with induction of autophagic vesicles; open arrowheads mark apoptotic cells. Cellular necrosis is also evident (circled cells) given increasing concentrations of C-PAC, particularly in OE19 cells (All images, 400X).

We previously determined the IC_50_ of C-PAC to be 50-100 μg/ml based on WST-1 and BrdU *in vitro* assays conducted in EAC (JHAD1 and OE19), lung (NCI-H460, misidentified as SEG-1) and colon (SW460, misidentified as BIC-1) cancer cell lines [16–18]. The latter two cell lines were accepted to be EAC cell lines for decades, but in 2010 DNA finger printing confirmed SEG-1 and BIC-1 to be of lung and colon origin, respectively [32]. The present study is the first to utilize authenticated human EAC cell lines and EAC xenografts to investigate cancer inhibitory mechanisms associated with C-PAC treatment. As illustrated in Figure 1A–1D and Supplemental Figure 1S, flow cytometric results from PI staining alone showed that C-PAC treatment of EAC cells resulted in a dose and time-dependent effect on phase of cell cycle. C-PAC [50 and 100 μg/ml] treatment of OE19 cells significantly decreased the percentage of G_1_ cells and significantly increased the percentage of cells at the G_2_-M checkpoint. A similar significant pattern of reduced G_1_ and increased accumulation of cells at G_2_-M was noted for C-PAC treated OE33 and JHAD1 EAC cells (Supplemental Figure 1S). Additionally, C-PAC [50 and 100 μg/ml] treatment of OE19 cell lines resulted in significantly increased S-phase fraction based upon PI staining alone (Figure 1A and 1C); thus, PI in combination with S-phase specific BrdU staining was conducted to assess S-phase distribution. BrdU incorporation plots by treatment are shown in Figure 1B for OE19 treated cells and Supplemental Figure 1S and Figure 1C for OE33 cells. Vehicle treated OE19 cells exhibited the highest intensity of BrdU staining corresponding to the highest proliferative rates, 66.9% compared to significantly reduced levels (14.4% and 0.4% BrdU) in OE19 cells treated with 50 and 100 μg/ml C-PAC, respectively. C-PAC significantly inhibited BrdU incorporation in a dose-responsive manner; slow proliferating cells represented 9.4% of the S-phase fraction in vehicle treated OE19 cells compared to 29% and 78% in 50 and 100 μg/ml C-PAC treated cells, respectively. Similarly, the percentage of OE33 cells in S-phase were significantly reduced by C-PAC, but without an S-phase delay (Supplemental Figure 1S and Figure 1C). Furthermore, DNA histogram results (Figure 1C) revealed that C-PAC induced a significant sub G_1_ peak (17.3%) characteristic of late apoptosis compared to only 1.8% in vehicle treated cells. Figure 1D depicts C-PAC induced changes in EAC cell morphology and illustrates reduced viability post-treatment as previously reported [18]. Characteristic features of cell death evident following C-PAC treatment included nuclear fragmentation and clumping, cellular blebbing, apoptotic residual bodies, but also cytoplasmic swelling with intact membranes and increased cytoplasmic vacuolization in JHAD1 and OE33, leading us to evaluate autophagy linked cell death. Cellular necrosis was evident given increasing concentrations of C-PAC, particularly in OE19 cells.

### C-PAC differentially induced cell death based on cell line acid resistance

**Figure 2:**
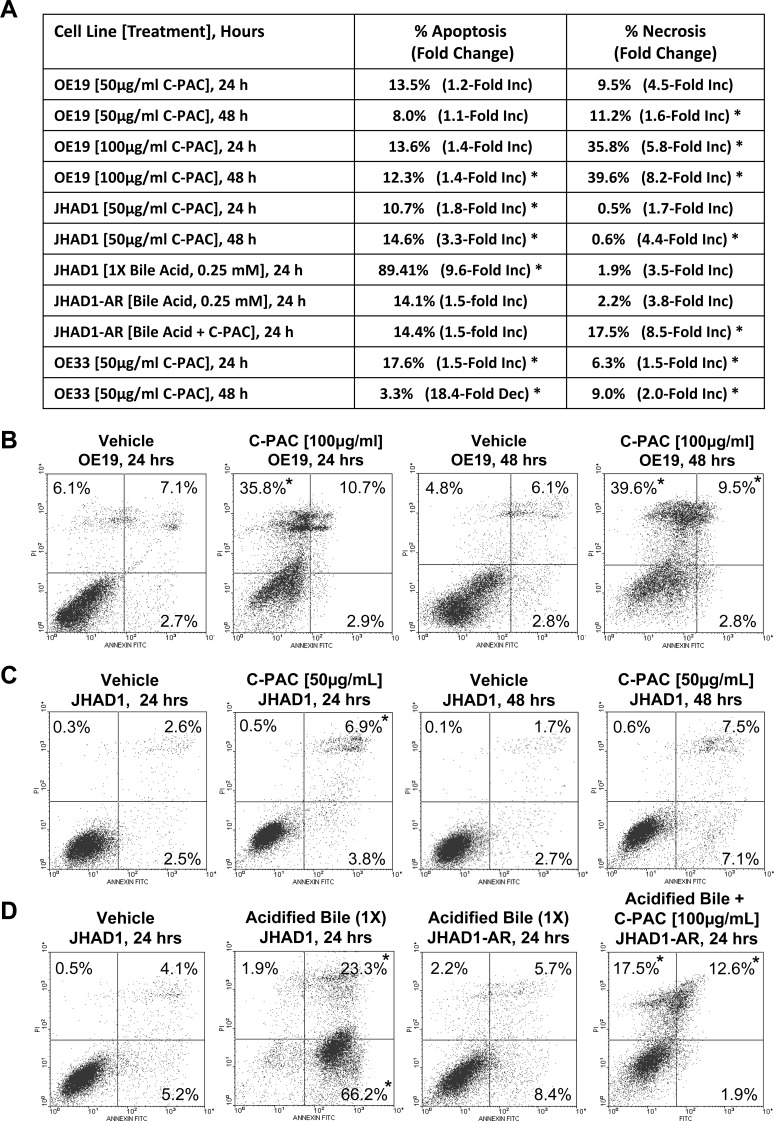

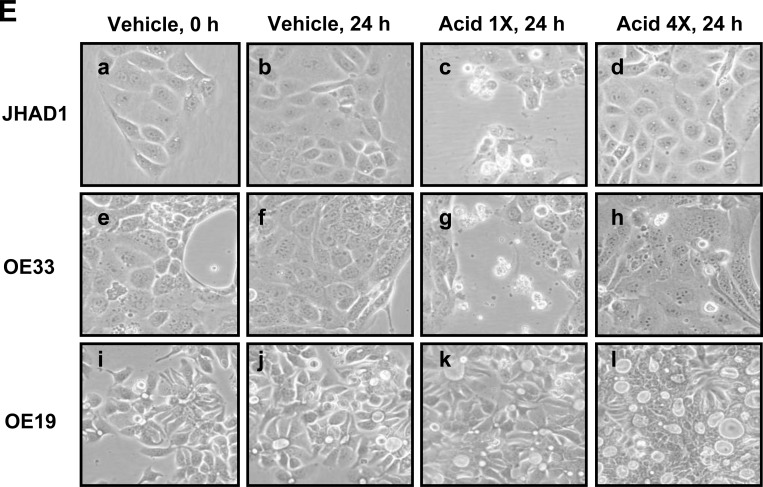
C-PAC differentially induced EAC cell line death based on acid-resistance EAC cells were treated with C-PAC [50 or 100 μg/ml] and harvested at 24 or 48 hours post-treatment for assessment of cellular apoptosis and necrosis using Annexin V-FITC staining coupled with flow cytometry. Data are represented as mean values from 3-4 independent samples and the asterisk (*) indicates a significant difference (*P* < 0.05) compared to vehicle treated cells. **A.** Summarizes the dose dependent and temporal effects of C-PAC on total apoptosis and necrosis induction in OE19, OE33 and naïve as well as acid resistant (AR) JHAD1 cells. Early and late apoptotic events were summed and data presented as a mean percentage of total apoptosis or necrosis with fold change from vehicle cells in parenthesis. **B.** Representative plots of Annexin V-FITC stained OE19 cells treated with vehicle or C-PAC [100 μg/ml] for 24 or 48 hours. **C.** Representative plots of Annexin V-FITC stained JHAD1 cells treated with vehicle or C-PAC [50 μg/ml] for 24 or 48 hours. **D.** Representative plots of Annexin V-FITC stained JHAD1 cells treated one time with an acid bile cocktail (1X), or multiple times (4X) developing acid resistant (AR) JHAD1 cells which were then treated with vehicle or C-PAC [100 μg/ml] for 24 hours. In B-D, the upper left, upper right and lower left quadrants represent necrotic, late apoptotic and early apoptotic events, respectively. Similarly, Supplemental Figure 1S, D shows C-PAC induced cell death effects in OE33 cells. **E**. Representative photomicrographs (400X) illustrates that a single exposure to an acidified bile cocktail results in rapid cell death of JHAD1 and OE33 cells (c and g), but not OE19 cells (k). Acid sensitive JHAD1 and OE33 cells become resistant to the acidified bile cocktail with repeated exposure (d and h).

Annexin V-FITC/PI staining of C-PAC [50 and 100 μg/ml] treated OE19, OE33 and JHAD1 cells harvested at 24 and 48 hours permitted assessment of cell death induction via apoptosis and necrosis as summarized in Figure 2A–2D and Supplemental Figure 1S, C. C-PACs [100 μg/ml] main effect in OE19 cells was markedly induced necrosis, 36% or 5.8-fold at 24 hours and 40% or 8.2-fold at 48 hours (*P* < 0.05); yet, only mild apoptosis was induced (12.3%, *P*<0.05). C-PAC [50 μg/ml] significantly induced apoptosis and to a lesser extent necrosis in OE33 cells at 24 and 48 hours post-treatment. C-PAC [50 μg/ml] induced significant apoptosis in JHAD1 cells at 24 and 48 hours.

We evaluated how EAC cell lines responded to a single 5 minute exposure to an acidified bile cocktail (pH = 4). Figure 2E shows that JHAD1 and OE33 cells were acid sensitive responding with rapid cell death (50 and 40%, respectively) 24 hours post-treatment. In sharp contrast, bile cocktail treated OE19 cells did not result in significant cell death induction, supporting inherent acid resistance compared to JHAD1 and OE33 cells. Next, JHAD1 and OE33 cells were repeatedly treated with the acidified bile cocktail every other day for 7.5 minutes mimicking multiple reflux episodes, as frequently occurs in patients with GERD. Following the fifth treatment both cell lines developed resistance based upon growth and confluency compared to naïve cells. Subsequently, acid sensitive or naïve JHAD1 cells and JHAD1 acid resistant (AR) cells were treated with C-PAC and evaluated for death inducing effects. Interestingly, upon a single 5 minute acidified bile cocktail treatment naïve JHAD1 cells experienced rapid and significant apoptosis. Early and late apoptosis levels were significantly increased to 59.6 and 3.7% at 6hrs; 53.7 and 31.9% at 12 hours; and 66.2 and 23.2% at 24 hours, respectively (*P* < 0.05 compared to vehicle treated cells). Conversely, when JHAD1-AR cells were acid-pulsed cell death was not significantly induced (Figure 2D); however, C-PAC treatment of JHAD1-AR cells resulted in significantly increased cell death via late apoptosis (12.6%) and to a greater magnitude cellular necrosis, 17.5% or 8.5-fold, similar to C-PAC induced death in constitutively acid resistant OE19 cells.

### C-PAC induced autophagy

**Figure 3:**
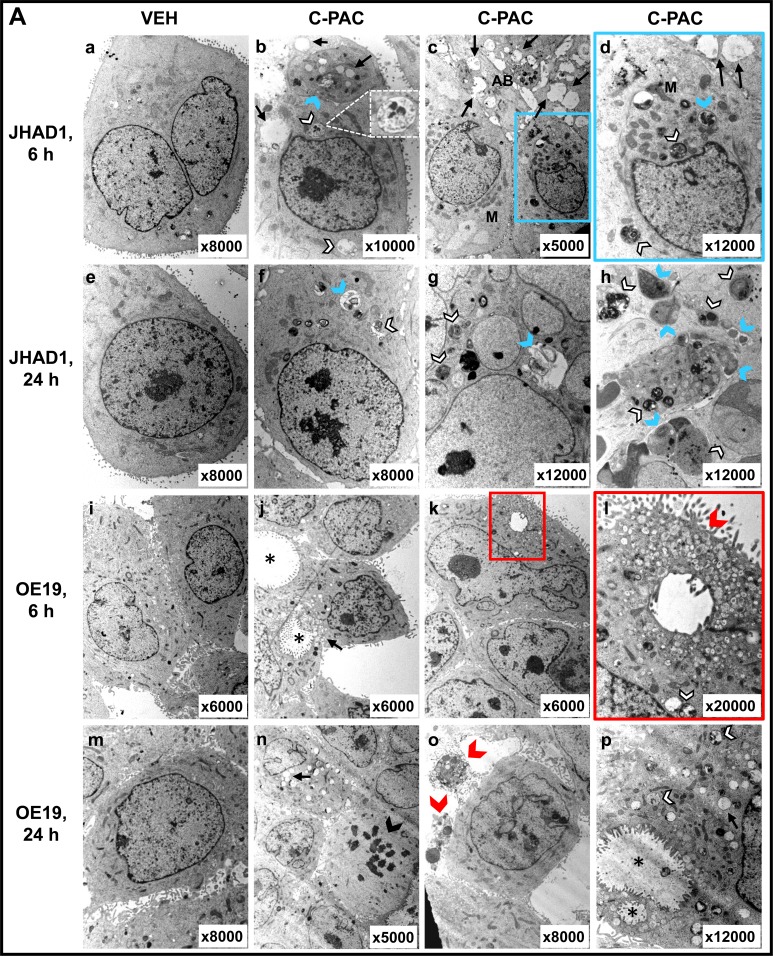

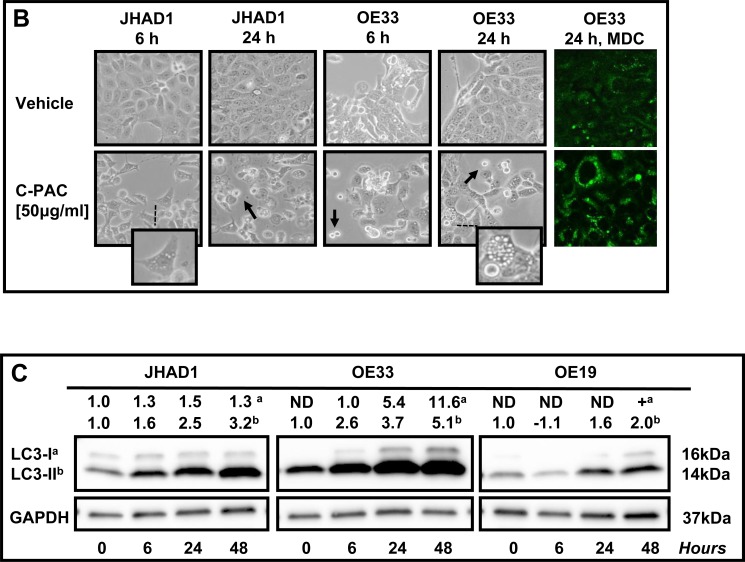
Effect of C-PAC on autophagy induction in EAC cells **A.** Ultrastructural changes associated with C-PAC treatment of EAC Cells. Transmission electron micrographs were captured of JHAD1 and OE19 cells following 6 and 24 hours of treatment with C-PAC [50μg/ml] to evaluate autophagic vacuole formation. a) and e) show vehicle treated JHAD1 cells; whereas, i) and m) show vehicle treated OE19 cells at 6 and 24 hours, respectively. Vehicle treated cells are characterized by microvilli protruding from the cell surface, a round smoothly outlined normal appearing nuclei, normally distributed heterochromatin, well defined plasma membrane and well preserved cytoplasmic organelles. b), c), and d) show cytoplasmic vacuolization (black arrows), increased formation of single and double walled autophagic vesicles as shown as autophagosomes (blue arrowheads) and autolysomes (white arrowheads, black outline) as early as 6 hours post C-PAC treatment of JHAD1 cells. Increased autophagy continues 24 hours following C-PAC treatment as displayed in f), g), and h) with increased electron dense cargo supporting formation of degradative autophagic vacuoles. C-PAC treatment of OE19 cells induced cellular necrosis as illustrated in j) and k) by moderate chromatin clumping and nuclear disintegration or karyolysis (asterisk), accompanied by loss of plasma membrane integrity and marked loss of cellular contents as in k), l), and o) (extra-cytoplasmic cellular debris, red arrowhead). Cellular blebbing (k, n) and fragmentation of the nucleus (Karyorrhexis, black arrowhead) are evident. p) Autophagic vacuoles containing cellular debris indicate a low level of autophagy induction in OE19 cells. **B.** Representative photomicrographs (200X) of JHAD1 and OE33 EAC cells treated with C-PAC [50 μg/ml] for 6 and 24 hours, black arrows indicate apoptotic cells. The enlarged inset illustrates morphological changes of cytoplasmic swelling and vacuolization consistent with autophagy induction (as also noted in Figure 1D). Monodansylcadaverine (MDC) staining and confocal microscopy were employed next to probe autophagic vacuoles in OE19, JHAD1 and OE33 cells following C-PAC treatment [50 μg/ml] for 24 hours. C-PAC treatment of OE33 cells resulted in increased accumulation of punctate staining as illustrated in large MDC positive vesicles (far right, bottom panel). **C.** To further investigate C-PAC induced autophagy, JHAD1, OE33 and OE19 cells were treated with C-PAC [50 μg/ml] or vehicle in triplicate and lysates collected at baseline and following 6, 24 and 48 hours of treatment; then, probed for LC3B, microtubule-associated protein 1 light chain 3 beta, an important early marker and effector of autophagy. Representative images are shown, expression values were normalized to the loading control GAPDH and a mean fold change from baseline or time of first detection calculated utilizing Quantity One software (Bio-Rad, Hercules, CA). Positive fold change values indicate increased expression and negative values reflect decreased expression. Figure 3C shows that C-PAC strongly (*P* < 0.05, *t-*test) induces the autophagy-associated lipidated form of LC3-II in JHAD1 and OE33 cells at 24 h and 48 hours; conversely, C-PAC treatment of OE19 cells modestly increased the autophagic form of LC3 and non-lipidated LC3-I levels were not detected until 48 hours.

TEM, MDC staining, and specific autophagic proteins were examined next to evaluate C-PAC as an inducer of autophagy in EAC cells. TEM, considered the gold standard for autophagy, permitted assessment of C-PAC induced ultrastructural changes in OE19 and JHAD1 cells (Figure 3A). Vehicle treated cells displayed normal morphology characterized by round smoothly outlined appearing nuclei, normally distributed heterochromatin, well defined plasma membrane and preserved cytoplasmic organelles. In contrast, C-PAC treated JHAD1 cells (Figure 3A, b-d) showed cytoplasmic vacuolization and increased early (6 hours) formation of double and single walled autophagic vesicles. Increased autophagy continued in C-PAC treated JHAD1 cells at 24 hours (f-h) with increased electron dense cargo supporting formation of degradative autophagic vacuoles. C-PAC treated OE19 cells exhibited features of cellular necrosis as illustrated in j) and k) by moderate chromatin clumping and nuclear disintegration, accompanied by loss of plasma membrane integrity and marked loss of cellular contents (k, l), and extra-cytoplasmic cellular debris (o). Cellular blebbing (k) and karyorrhexis (n) are also evident. Photomicrographs in Figure 3B (400 X) further illustrate the rapid changes in cellular morphology following C-PAC treatment of JHAD1 and OE33 cells. JHAD1 cells treated with C-PAC showed the formation of elongated spindle shaped cells and increased cytoplasmic extensions, early cytosolic vacuolization (enlarged inset), cytoplasmic swelling, yet intact nuclei in subpopulations of cells. The far right panel of Figure 3B shows a representative MDC stained image of OE33 cells treated with vehicle (top) and C-PAC 50 μg/ml] (bottom). Fluorescent MDC staining shifted from a generalized weak and diffuse pattern in vehicle treated cells to a pattern of significant cell enlargement and markedly increased accumulation of intense punctate MDC positive vesicles 24 hours post C-PAC treatment [50 μg/ml] consistent with autophagy induction. A similar pattern was noted in JHAD1 cells, but not OE19 cells (data not shown).

To further investigate C-PAC induced autophagy, JHAD1, OE33 and OE19 cells were treated with C-PAC [50 μg/ml] or vehicle and lysates collected at baseline and following 6, 24 and 48 hours of treatment; then, probed for LC3, microtubule-associated protein 1 light chain 3 beta, an important early marker and effector of autophagy. LC3 is modified via an ubiquitylation-like system generating a soluble form, LC3-I, which in turn is modified during autophagy induction to a membrane-bound form, LC3-II. Starting at 6 hours, C-PAC strongly induced the autophagy-associated lipidated form of LC3-II in JHAD1 and OE33 cells (Figure 3C *P* < 0.05, *t*-test) with increased magnitude at 24 and 48 hours (2.5 to 5.1-fold) supporting early autophagosome formation. Conversely, C-PAC treated OE19 cells modestly increased the autophagic form of LC3 (1.6 to 2.0 fold, *P* < 0.05) and non-lipidated LC3-I levels were not detected until 48 hours suggesting altered autophagy machinery and differing constitutive expression levels across EAC cell lines.

### Effect of C-PAC on cell-cycle, P53, PI3K/AKT/mTOR, p38 MAPK and cell-death associated proteins in EAC cells

**Figure 4:**
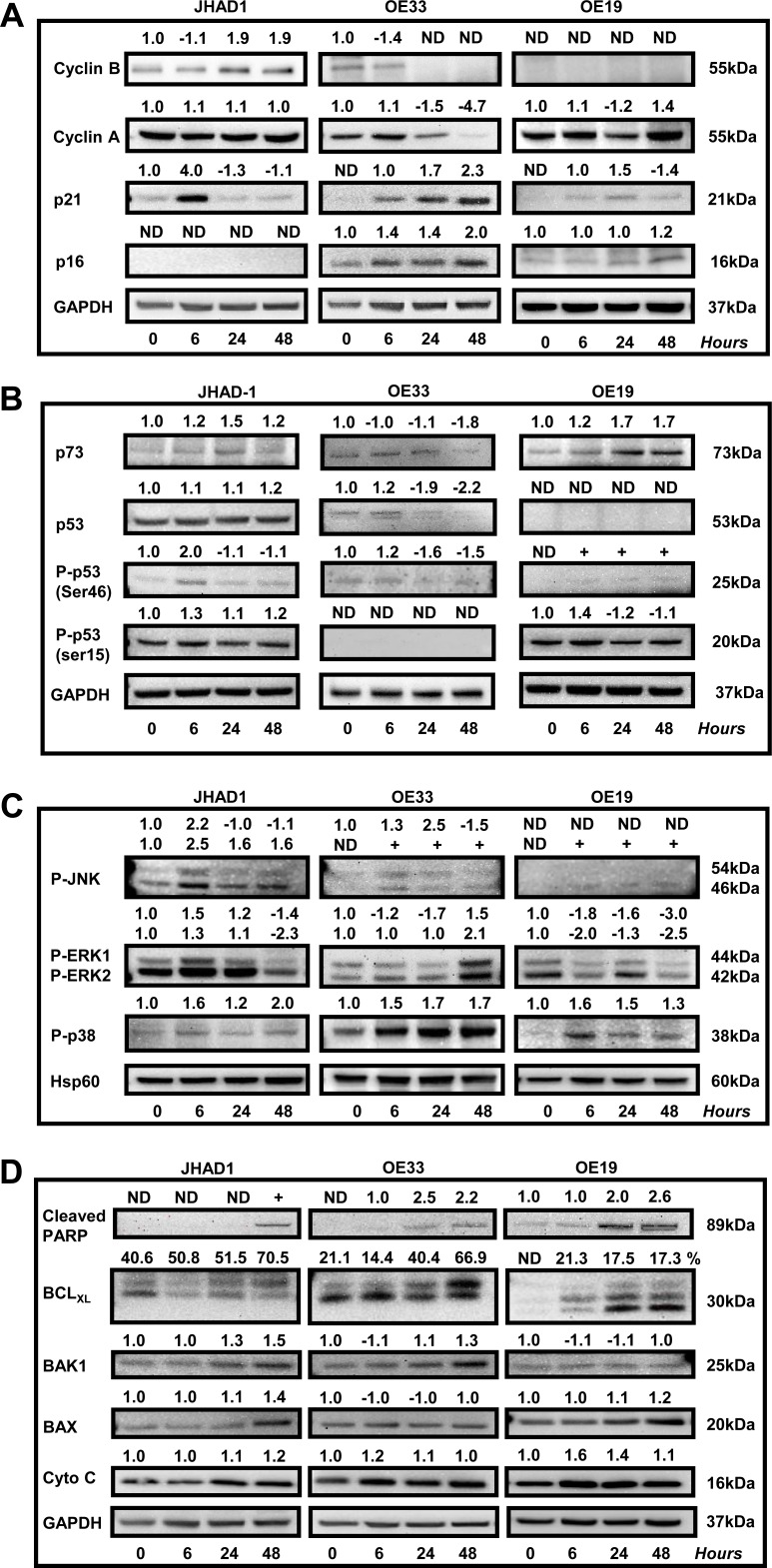

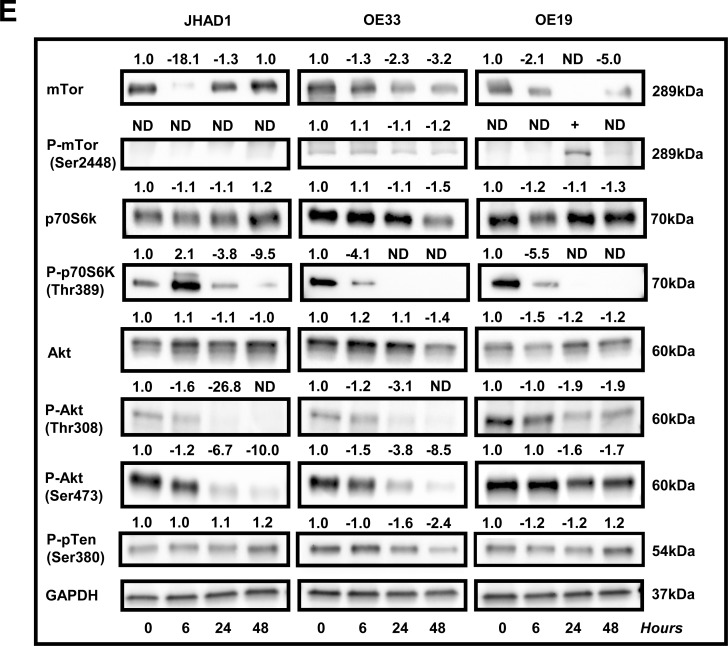
Effect of C-PAC on cell-cycle, P53, PI3K/AKT/mTOR, p38/MAPK and cell-death associated proteins in EAC cell lines JHAD1, OE33 and OE19 EAC cells were treated with C-PAC [50 μg/ml] or vehicle and lysates isolated at pretreatment or baseline and at 6, 24 and 48 hours following treatment. Immunoblot was performed using commercially available antibodies to proteins of interest. Expression values were normalized to the appropriate loading control, GAPDH or HSP60, and a fold change from baseline or first detection calculated based on two independent experiments. Positive fold change values indicate increased expression and negative values reflect decreased expression, except for BCL-xL. The top or deamidated BCL-xL band was quantified as a percentage of the total species to differentiate between the molecules pro- and anti-apoptotic characteristics. Effects of C-PAC treatment on the expression of a panel of **A.** cell-cycle regulatory molecules; **B.** P53 family members; **C.** MAPK signaling molecules; **D.** Pro- and anti-apoptotic markers; and **E.** PI3K/AKT/mTOR signaling molecules.

JHAD1, OE33 and OE19 were treated with C-PAC [50 μg/ml] or vehicle and lysates isolated at 0, 6, 24 and 48 hours. As shown in Figure 4A–4E, EAC cell lines were assessed for temporal changes in the expression of proteins involved in cell cycle (Cyclin A1, Cyclin B1, P16, P21), P53 signaling (P53, P73, P-P53 at SER46 and SER15), kinase signaling (P-JNK, P-ERK, P-P38), PI3K/AKT/mTOR (mTOR, P-mTOR, p70S6K, P-p70S6K, AKT, P-AKT^Thr308^, P-AKT^Ser473^, P-pTen) apoptosis (cleaved PARP, BCL-xL, BAK1, BAX, Cytochrome C, Caspases 3,4,7,8,9) and autophagy (LC3B). Expression values were normalized to the appropriate loading control and a fold-change from baseline or first detection calculated.

Considering C-PAC induced changes in cell cycle distribution we measured effects on levels of select cell cycle regulatory proteins (Figure 4A). Cyclin A1 regulates both the G_1_-S and G_2_-M checkpoints, whereas Cyclin B1 is a regulator of mitosis. Cyclin A1 levels were essentially unchanged in C-PAC treated JHAD1 cells; markedly reduced in OE33 cells (1.5-fold and 4.7-fold at 24 and 48 hours); and in OE19 cells, levels were transiently decreased followed by a 1.4-fold increase at 48 hours. Levels of cyclin B1 were increased in JHAD1 C-PAC treated cells (1.9-fold, 48 hours); whereas, levels declined to undetectable in OE33 cells following C-PAC treatment (24 and 48 hours). Cyclin B1 levels were not detected in OE19 cells at baseline or following treatment. C-PAC treatment increased expression levels of the tumor suppressor, P16 in a time-dependent manner in only OE33 cells (1.4 fold at 6 and 24 hours, 2.0 fold at 48 hours) which is promising considering its linkage to EAC progression. Levels of P16 were unchanged in OE19 cells and undetectable in JHAD1 cells where it is known to be deleted. Next, P21 was evaluated given its role in diverse cell cycle regulatory function at both G_1_ and G_2_-M checkpoints. Although basal levels of P21 were low in all EAC cell lines, C-PAC treatment increased levels showing an early, but transient 4.0-fold level increase in JHAD1 cells and increased levels in OE19 cells at 6 and 24 hours. Whereas, OE33 cells showed the highest magnitude and sustained increase in P21, changing from non-detectable levels at baseline to 1.7- and 2.3-fold increased levels at 24 and 48 hours, respectively. Also linked with p21 and cell cycle regulation is the critical tumor suppressor protein P53 which is inactivated early, frequently and linked to poor prognosis for EAC [33, 34] and is now known to be mutated in all three EAC cell lines evaluated [35]. JHAD1 cells expressed P53 in all forms and C-PAC induced a 2-fold increase in pro-apoptotic P-p53^ser46^ and an increase in P-p53^ser15^ at 6 hours; whereas, in OE19 cells C-PAC induced strong expression of only P-p53^ser15^ which is linked to DNA damage induced cell death. JHAD1 cells reportedly have a somatic mutation in exon 8 resulting in a non-synonymous Gly 266 Glu alteration [36]. Still, in JHAD1 cells, C-PAC induced cell death may be partially p53-mediated as the p53 target BAX is also up-regulated (1.4 fold, 48 hours). OE19 cells are reported to have partially functional p53, despite showing a total lack of P53 expression and very low P-P53^ser46^ expression. OE33 cells have a p53 mutation in exon 5, resulting in non-functional nuclear protein accumulation and weak expression of P-P53^ser46^. The p53 homolog p73, known to mediate apoptosis and autophagy via the mTOR pathway [36, 38] was also evaluated. C-PAC treatment resulted in a consistent temporal increase (6-48 hours) in P73 expression levels in OE19 and JHAD1 cells, but P73 levels in C-PAC treated OE33 cells were unchanged followed by a reduction at 48 hours.

Mitogen-activated protein kinases (MAPKs) are a family of serine-threonine kinases that constitute a dominant signaling pathway in esophageal adenocarcinoma progression and have an essential role in signal transduction, cellular growth, proliferation, migration and death [39]. MAPKs are comprised of extracellular signal regulated kinases (ERKs), c-Jun *N-*terminal kinases (JNKs) and p38 MAPKs, each of which we evaluated in EAC cells following C-PAC treatment (Figure 4C). JNK phosphorylation was generally increased in EAC cells by C-PAC treatment; however, the level, temporality and magnitude differed across cell lines. More striking expression changes were noted in both P-ERK1 (44 kD) and P-ERK2 (42 kD). C-PAC treated JHAD1 cells showed increased P-ERK1/2 levels at 6h (1.5/1.3-fold, respectively). In contrast, P-ERK1/2 declined in a temporal manner in treated OE19 cells starting at 6 hours, with greatest effects at 48 hours. In OE33 cells, C-PAC treatment decreased levels of P-ERK1 at 6 and 24 hours (1.2 and 1.7-fold) followed by 2.1-fold increased P-ERK2 levels at 48 hours. C-PAC increased levels of P-p38, a marker generally thought to have a tumor suppressive role. JHAD1 cells showed a biphasic change in P-p38 in response to treatment (1.6, 1.2-and 2.0-fold increased levels at 6, 24 and 48 hours, respectively). P-p38 levels were strongly expressed in OE33 cells and increased in a time-dependent manner from 1.5-fold at 6 hours to 1.7-fold at 48 hours. Treated OE19 cells showed increased (1.6-fold) P-p38 at 6 hours, followed by gradual reductions through 48 hours. Our data support differential activation of MAPK molecules by C-PAC temporally and in a cell-line specific manner.

Considering C-PACs ability to induce apoptosis, autophagy and necrosis, a host of key cell death signaling molecules were evaluated. PARP cleavage is considered a hallmark of apoptosis induction, but is also implicated in DNA repair and DNA-damage induced autophagy [40–42]. All EAC cell lines showed a time-dependent increase in PARP cleavage after C-PAC treatment, with the strongest and earliest induction noted in OE19 cells (Figure 4D). However, C-PAC treatment failed to activate effector caspase 7 in EAC cells (Supplemental Figure 2S) and caspase 3 cleavage was weak, only detected with extended exposure times. In addition, C-PAC had little effect on initiator caspases 8 or 9 or caspase 4, involved in inflammatory cytokine processing. C-PAC induced expression of pro-apoptotic BCL2 members, BAX and BAK in JHAD1 cells (48 h) and increased BAK in OE33 cells (48 h). Additionally, the phosphorylated form of BCL2, which acts to suppress the anti-apoptotic effects of BCL2, was modestly elevated only in OE19 cells at 24 and 48h (data not shown), but not detectable in JHAD1 or OE33 cells. C-PAC treatment increased levels of the pro-apoptotic deamidated form of BCL-xL [43, 44], in a time-dependent manner as indicated by the percentage comprising the upper band of BCL-xL (Figure 4D), in both JHAD1 (40.6-70.5%) and OE33 (14.4-66.9%) cells. In contrast, OE19 cells showed increased expression of deamidated BCL-xL, but the magnitude was much lower (17.3-21.3%) supporting greater resistance.

As shown in Figure 4E, C-PAC treatment strongly inactivated PI3K/AKT/mTOR signaling networks as supported by total loss or significant reduction of P-p70S6k, P-Akt^Thr308^ and P-Akt^Ser473^ expression in all EAC cell lines 24 and 48 hours post C-PAC treatment. mTOR levels also decreased following C-PAC treatment. Collectively, the results support C-PAC inhibition of mTORC1 and mTORC2 signaling. P-pTEN was evaluated given its role in the regulation of PI3K/AKT signaling and autophagy induction. C-PAC markedly reduced levels of P-pTEN at 24 and 48 hours (1.6 and 2.4-fold, respectively) in OE33 cells; resulted in minor reductions in OE19 cells, but had little impact in JHAD1 cells indicating a cell line specific effect between pTEN dephosphorylation and AKT inactivation.

### C-PAC inhibited growth of OE19 tumor xenografts by modulation of cell cycle, PI3K/AKT/mTOR and MAPK signaling pathways

**Figure 5:**
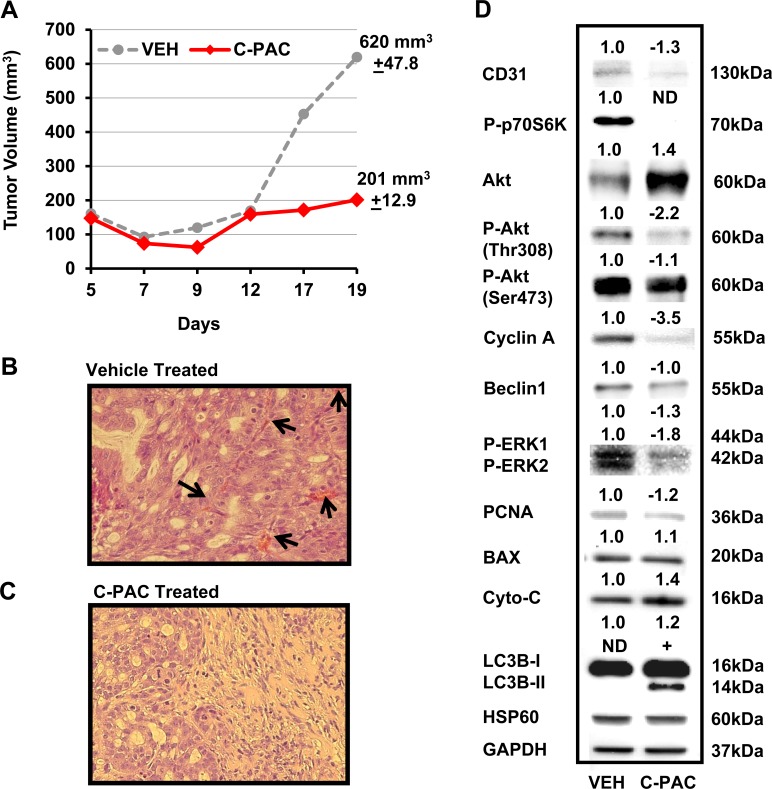
Effect of C-PAC treatment on OE19 xenografts in athymic NU/NU mice Mice were implanted s.c. with 1.25 × 10^6^ OE19 cells in each flank. Tumors grew for 5 days reaching a mean tumor volume of 150 mm^3^ prior to mice being randomized into vehicle or C-PAC treatment groups. C-PAC was delivered by oral gavage at a concentration of 250 μg/mouse, 6 days a week. Increasing tumor size in the vehicle treated groups required termination of the experiment on day 19. Data are shown as mean percentages + SEM. *P < 0.05 indicates a significant difference between C-PAC and vehicle treated mice, two-tailed Students *t*-test. **A.** C-PAC administration significantly inhibited mean tumor volume by 67.5% compared to tumors in vehicle treated mice. **B.** Vehicle treated tumors had greater cellular pleomorphism, inflammation (lower left) and markedly increased blood vessel formation as evidenced by areas of bright eosinophilia (black arrows) **C.** C-PAC treated tumors displayed areas of relatively normal epithelium as well as glandular precursor lesions, similar to Barrett's esophagus. **D.** Protein lysates isolated from C-PAC treated OE19 xenografts showed alterations in AKT/mTOR/MAPK signaling and specific markers linked to cell cycle progression, apoptosis, and autophagy compared to vehicle treated xenografts.

On the basis of our positive *in vitro* results supporting C-PAC has cancer inhibitory potential in EAC cells; we next evaluated the *in vivo* tumor inhibitory potential of C-PAC utilizing OE19 xenografts in an athymic NU/NU mouse model. Unlike OE33 and JHAD1 cells, OE19 cells readily formed xenograft tumors reaching 150 mm^3^ 5 days post-injection of 1.25E6 OE19 cells per flank. On day 5, mice were randomized to receive vehicle or C-PAC [250 μg/day] via gavage 6 x weekly. Figure 5A–5D summarizes C-PACs effects on OE19 tumor growth, tumor morphology and alterations in the expression levels of cell cycle, proliferative, apoptotic, and AKT/mTOR/MAPK signaling proteins. C-PAC treatment did not alter body weight or food consumption (data not shown) in treated mice. Orally delivered C-PAC [250 μg/day] resulted in a significant, 67.6%, decrease in xenograft tumor volume. Figure 5B, illustrates that vehicle treated tumors had increased blood vessel formation as evidenced by areas of bright eosinophilia (black arrows). Whereas, C-PAC treated xenografts (C) displayed large areas of relatively normal epithelium mixed with focal glandular precursor lesions. Lysates isolated from C-PAC treated OE19 xenografts (D) showed decreased expression in the proliferative marker PCNA and to a greater extent cyclin A; cyclin B1 was not detected (data not shown). C-PAC treatment resulted in increased levels of pro-apoptotic cytochrome C (1.4-fold) and reduced P-ERK levels in C-PAC treated tumors, in line with decreased levels detected in C-PAC treated OE19 cells. C-PAC treatment also induced dephosphorylation of p70S6K, AKT^Thr308^, and reduced P-AKT^Ser473^ paralleling *in vitro* results. Lastly, C-PAC induced expression of autophagy associated LC3B-II in OE19 xenografts, but not Beclin-1 levels. Neither P-pTen nor activated caspases (3, 7 or 9) were detected in C-PAC treated xenograft lysates.

## DISCUSSION

Esophageal adenocarcinoma represents a significant health problem characterized by rapidly rising incidence, substantial morbidity and high mortality. Barrett's esophagus is the only recognized precursor lesion and although BE patients are successfully treated to relieve symptoms associated with acid reflux, none of the current therapies have proven inhibitory against EAC development. Considering the majority of BE patients are relatively healthy and do not progress to EAC [45] identification of non-toxic cancer inhibitory agents, acceptable for long-term administration, are needed. A number of bioactive constituents derived from plant or food-based sources have favorable toxicity profiles and hold promise as cancer inhibitors. Cranberry constituents have been shown to have strong anti-proliferative and apoptosis inducing capacity in a number of cancer cell lines and in a limited number of *in vivo* models [15–25]; however, pathways of inhibition are incompletely understood. Thus, the current study evaluated C-PAC induced EAC cell death mechanisms *in vitro* and *in vivo* to improve our understanding of targetable molecular pathways and the context in which EAC inhibition is permissive.

**Figure 6:**
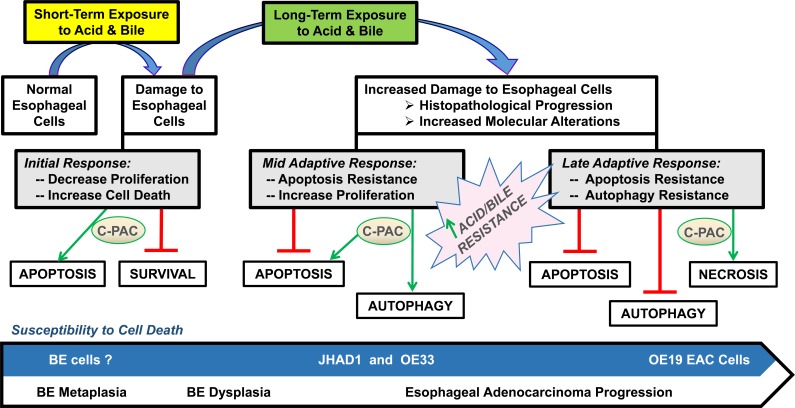
Proposed model for C-PAC induced cell death in EAC cell lines Results support C-PAC induces cell death by different mechanisms in EAC cell lines based on sensitivity to an acidified bile mixture. Exposing acid sensitive JHAD1 and OE33 EAC cells to an acidified bile cocktail (5 min, pH = 4.0) resulted in rapid cell death via early apoptosis; however, exposure of inherently acid resistant OE19 EAC cells does not result in significant cell death. C-PAC treatment of JHAD1 and OE33 cells results in cell death via low levels of apoptosis and significant autophagy; whereas C-PAC treated OE19 cells die largely by cellular necrosis and exhibit low levels of autophagy and apoptosis. Similarly, when JHAD1 cells are pushed to become resistant to the acidified bile cocktail (4-6 repeat exposures) the cells die via necrosis supporting that mode of cell death induction is linked to sensitivity to an acidified bile cocktail.

Results of the current study demonstrated that C-PAC has potent EAC cell death inducing capacity and strongly inhibits growth of EAC tumor xenografts. The research results presented appear to be the first to demonstrate C-PAC induced autophagy resulting in cancer cell death and the first to report *in vivo* efficacy of orally delivered C-PAC against EAC. Oral administration of C-PAC resulted in significantly reduced (68%) OE19 xenograft volume linked to markedly increased formation of autophagic LC3B-II and inactivation of PI3K/AKT/mTOR signaling as evidenced by dephosphorylation of p70S6K, AKT^Ser473^ and to a lesser extent AKT^Thr308^. Interestingly, autophagy induction *in vitro* and *in vivo* appeared to be Beclin-1 independent; also, reported to occur with treatment of resveratrol [46] and *cis*-unsaturated fatty acids [47]. Other effects noted in lysates from C-PAC treated mice include increased levels of pro-death BAX and Cytochrome C, reduced Cyclin A and PCNA, as well as reduced P-ERK1/2 supporting additional effects on MAPK signaling networks. Fitzgerald and colleagues identified the MAPK pathway to be highly induced (42.7%) following receptor tyrosine kinase activation (RTK) in esophago-gastric tumors [48]. Importantly, bile and/or acid exposure, well-documented risk factors for EAC, are linked to aberrant MAPK and PI3K/AKT/mTOR signaling [26–32, 48]. Also downstream of RTK activation is the PI3K/AKT/mTOR pathway regulating diverse cellular functions involved in EAC cancer progression including advanced tumor stage, poor prognosis and therapeutic resistance [49–53]. Hence, results are promising in that C-PAC mitigates aberrant PI3K/AKT/mTOR and MAPK signaling cascades *in vivo* and *in vitro*. Moreover, *in vitro* findings support that the mode of C-PAC induced cell death varies based on acid resistance of the EAC cells. C-PAC induced cell death via low levels of mainly caspase independent apoptosis and significant autophagy in acid-sensitive JHAD1 and OE33 EAC cells, but mainly through cellular necrosis in OE19 cells which exhibited greater constitutive resistance to acidified bile salt exposure, as well as resistance to apoptosis and autophagy induction, as summarized in Figure 6. The role of autophagy in EAC and BE is a poorly understood and understudied area to date. Dvorak and colleagues recently reported a significant decrease in expression of Beclin-1, a key autophagy regulator, in dysplastic BE and EAC cases compared to non-dysplastic BE tissues and normal squamous epithelium [54]. Interestingly, acute bile acid exposure increases Beclin-1 levels inducing autophagy; whereas, chronic or long-term bile acid exposure leads to a reduction in Beclin-1 expression and inhibition of autophagy [54]. Our results lend further support to autophagy induction as a pro-death mechanism in EAC, particularly in apoptosis resistant cells. Beclin-1 expression is linked to improved survival in patients with squamous cell cancer of the esophagus [55], but additional autophagy research is warranted in the context of EAC disease progression and prognosis given clinical interest in both autophagy inhibitors and inducers as therapeutic agents.

Cell line specific responses following C-PAC treatment were noted for select cell cycle and cell death associated markers; yet, common biological responses included G_2_-M cell cycle arrest. The strongest effects of C-PAC induced G_2_-M arrest were noted in OE33 cells, followed by OE19 and significant, but modest effects in JHAD1 cells. Cyclin A1 regulates both the G_1_-S and G_2_-M checkpoints, correlates with degree of dysplasia in BE patients and is linked to cancer invasiveness [52, 53]. Cyclin A1 levels were essentially unchanged in C-PAC treated JHAD1 cells, markedly reduced in OE33 cells (4.7-fold), transiently decreased in OE19 cells (1.2-fold), and markedly reduced (3.5-fold) in C-PAC treated xenografts. Cyclin B1, charged with transitioning cells from G_2_ to M, was not detected in OE19 cells; whereas, levels decreased 24 hours post-treatment in OE33 cells to below detection limits as is commonly reported with G_2_-M arrest and more specifically late G_2_ arrest. In contrast, C-PAC treated JHAD1 cells displayed increased Cyclin B1 levels (1.9-fold) suggesting M-phase arrest and potentially mitotic catastrophe resulting from DNA damage. DNA damage induced MAPK activation also contributes to p21 induction and cell cycle arrest. P21 is known to be involved in diverse cell cycle regulatory functions at both G_1_ and G_2_-M checkpoints. Interestingly, OE33 cells showed the highest magnitude and sustained increase in P21, changing from non-detectable at baseline to 2.3-fold increased levels at 48 hours. The latter finding is potentially linked to the fact that p21 levels peak during G_2_ and that C-PAC induced the highest magnitude of G_2_-M arrest in OE33 cells.

In terms of cell death associated molecules, C-PAC treated EAC cells responded with increased cleaved PARP and cytochrome C levels, but effects were generally caspase independent, with only low level induction of activated caspase 3 (Supplemental Figure 2S). Caspase-independent PARP cleavage can lead to apoptotic or necrotic cell death and our data suggests a role for autophagy induction as well. A novel unexpected finding was that C-PAC induced deamidation of BCL-xL, a pro-death form of the important pro-survival BCL-2 family member protein and one whose functional consequences are still being unraveled [43, 44, 56]. However, recent research suggests factors decreasing BCL-xL deamidation may increase tumor cell viability and increase resistance to cancer treatment [56]. In brief, Weintraub and colleagues revealed that DNA damaging agents induce BCL-xL deamidation of two asparagines by disrupting the capacity of BCL-xL to inhibit the proapoptotic activity of BH3 domain-only proteins; in turn, increasing apoptosis susceptibility [42]. Results herein suggest the first time that BCL-xL deamidation may also play a role in autophagy induction in apoptotic resistant EAC cells.

Our data provide novel insight into mechanisms of C-PAC induced cell death *in vitro* and *in vivo* providing the first evidence that C-PAC activated autophagy serves as an alternative cell death pathway in apoptotic and acid resistant EAC cancer cells; yet, C-PAC targeted necrosis in apoptosis and autophagy resistant cells. C-PACs pro-death effects were accompanied by inactivation of PI3K/AKT/mTOR signaling, modulation of MAPKs and a G_2_-M cell cycle arrest. Still, additional research is warranted to assess the effects of C-PAC on mitophagy and chaperone-mediated autophagy as well as ROS generation and DNA damage. The current study results lay the foundation and direction for future research utilizing genetic and pharmacologic approaches to further improve our understanding of C-PACs potential to target EAC or Barrett's esophagus. Additional research is ongoing to assess the effects of C-PAC utilizing normal esophageal cells and a panel of premalignant BE cell lines, as well as a rat model in which reflux-inducing surgery results in EAC. Future *in vivo* research should also consider evaluations of primary esophageal xenografts which have the advantage of preserving cancer stem cell populations, stromal components and greater functional characteristics of the primary cancer [57] and in turn may offer improved personalized preventive or therapeutic approaches.

## MATERIALS AND METHODS

### Cranberry extract isolation, purification and dose determination

Cranberry fruit (*Vaccinium macrocarpon* Ait.) was collected at the Marucci Center for Blueberry and Cranberry Research, Chatsworth, NJ. Purified C-PAC extract was isolated from cranberries of the ‘Early Black’ cultivar utilizing solid-phase chromatography according to well established methodology [9–12]. In brief, the fruit was homogenized in 70% aqueous acetone, filtered and the pulp discarded. Collected cranberry-derived proanthocyanidins were concentrated under reduced pressure and purified extract isolated using bioassay-directed fractionation. The absence of absorption at 360 nm and 450 nm confirm all but proanthocyanidins are removed. Additional methods including ^13^C NMR, electrospray mass spectrometry, matrix-assisted laser desorption/ionization time-of-flight mass spectrometry, and acid catalyzed degradation with phloroglucinol were utilized to verify the presence of A-type linkages as well as to determine the concentration of proanthocyanidins in the purified extract. C-PAC is comprised of five main proanthocyanidins as previously characterized by Dr. Howell and colleagues [11]. The proanthocyanidin molecules largely consist of epicatechin units with degrees of polymerization of 4 or 5, as well as epigallocatechin and catechin. C-PAC contains three types of linkages, two common B-type linkages (*C4→C6* and *C4→C8*) and at least one unique A-type ether linkage (*C2→O→C7*) found only in cranberry, chokeberry, plums and avocado [14, 15]. Purified C-PAC was freeze-dried and stored at −80°C. C-PAC concentrations chosen for study were informed by our earlier research which determined the LD50 to be in the 50 to 100 μg/ml range in various cancer cell lines [16–18]. Consideration was also given to earlier evaluations by Howell and colleagues showing 50 μg/ml of C-PAC inhibits adhesion of p-fimbriated uropathogenic E. coli bacteria *in vitro* and that 36 mg/day of C-PAC delivered in 10 ounces of juice inhibits bacterial adhesion in the urinary tract wall of humans [9–13]. Importantly, the concentrations of C-PAC under evaluation in this series of preclinical investigations are readily achievable in humans and are already under evaluation for oral and urinary tract health benefits.

### Cell culture

Authenticated human EAC cell lines utilized in this series of experiments [32] included JH-ESOAd1, referred to as JHAD1, isolated from a distal EAC, stage III, N0 in 1997 (Johns Hopkins University, Baltimore, MD); OE33 cells isolated in 1993 from a distal EAC, stage II, N0, (ECACC, Wiltshire, UK); and OE19 cells isolated in 1993 from an adenocarcinoma at the gastro-esophageal junction, stage III, N1 (ECACC, Wiltshire, UK). Cells were grown in RPMI 1640 complete medium containing L-glutamine (2.0 mM), penicillin (10^4^ units/mL), streptomycin (10^4^ μg/mL), sodium pyruvate (1 mM), and 5-10% fetal bovine serum depending on the experiment. Cells were maintained as monolayers at 37°C with 95% air and 5% CO_2_ for all studies.

### Determination of apoptosis, phase of cell cycle and BrdU levels by flow cytometry

OE19, JHAD1 and OE33 cells were plated in T25 flasks at 1.5E6, 1.0E6 and 1.0E6 cells, respectively and incubated for 24-30 hours prior to treatment with C-PAC [50 or 100 μg/ml] or vehicle [<0.001% ETOH] in phenol red free RPMI complete medium with 5% FBS. Each evaluation was performed in triplicate per experimental time-point. Cell cycle distribution was assessed at 24 and 48 hours utilizing propidium iodide (PI) detection (BD Sciences, Palo Alto, CA) as previously described [16–18]. Briefly, harvested cells were fixed in ice cold 70% EtOH, washed in PBS and stained with PI. A minimum of 20,000 cells were analyzed per treatment utilizing a FACSCalibur flow cytometer. ModFit LT software (Verity, Topsham, ME) was used to determine the percentage of cells in each phase of the cell cycle (G0-G_1_, S, G_2_-M). Concurrent BrdU [10μM, 1h incubation] and PI staining was conducted to improve our understanding of the S-phase distribution beyond that provided by PI-based analysis alone. BrdU distribution and intensity of staining was detected by FACSCalibur analyzing a minimum of 10,000 cells; whereas, Annexin V-FITC staining methods were employed to detect apoptotic and necrotic events counting a minimum of 10,000 cells with Flowjo software employed for analysis.

### Protein isolation and Western blot analysis

JHAD1, OE33 and OE19 EAC cells were plated in T75 flasks at 1.5E6, 1.5E6 and 3E6 cells, respectively. Cells adhered for 24-30 hours, were treated with C-PAC [50 μg/mL] or vehicle and harvested at 0, 6, 24 and 48 hours post-treatment. Cell lysates were prepared using Cell Signaling lysis buffer (Cell Signaling Technology, Inc., Beverly, MA.). Protein was quantified using the Quick Start Bradford protein assay and equivalent protein amounts loaded into precast NuPage Novex Bis-Tris 10% gels (Life Technologies, Carlsbad, CA.) and mini-protean TGX gels (4-20%, 7.5%, 10% and 12%; Bio-Rad). Immunoblotting was performed in duplicate or triplicate using commercially available antibodies from Santa Cruz Biotechnology (CD31, Cytochrome C, GAPDH, P16, P21, PCNA) and Cell Signaling (AKT, BAK1, BAX, BCL-xL, Beclin-1, Caspases 3/4/7/8/9, LC3B, Cyclin A1, Cyclin B1, PARP, P-AKT^Thr308^, P-AKT^Ser47^, P-BCL-2^Ser70^, P-ERK, P-JNK, P-pTEN, P-P38, P-p70S6K, P-mTOR, p70S6K, all P53 family member antibodies and P73) to proteins of interest. Expression values normalized to loading controls were determined by chemiluminescent immunodetection with fold-change from baseline or first appearance reported.

### Acidified bile challenge and development of acid/bile resistant cells

EAC cells were treated with a 0.2mM acidified bile cocktail (pH = 4.0) comprised of equal amounts taurocholic, glycocholic, glycodeoxycholic, glycocheno-deoycholic and deoxycholic acids (Sigma-Aldrich) mimicking human refluxant [27]. Cells received either a single 5 minute pulsatile treatment with an acidified bile cocktail or a single treatment, followed by multiple additional exposures of 7.5 minutes every other day to simulate repeated reflux episodes. Each cell line was evaluated in terms of constitutive acid sensitivity, capacity to develop acid resistance and whether resistance impacted mode of C-PAC induced cell death.

### Transmission electron microscopy

OE19 and JHAD1 cells were plated (2.5E4 and 1.2E4, respectively) in LabTek 4 well permanox chamber slides with RPMI complete medium and adhered for 30 hours prior to C-PAC [50 μg/ml] treatment. Cells were harvested at 6 and 24 hours post-treatment and fixed overnight in 2% glutaraldehyde in 0.1M phosphate buffer, pH 7.4 containing 0.1M sucrose, followed by dehydration in a graded series of acetone, infiltration with resin, embedding, and preparation of 80 nm sections and staining. Sections were examined and transmission electron images captured to assess the ultrastructural features and the formation of autophagic vacuoles.

### Monodansylcadaverine staining of autophagic vacuoles

EAC cells were plated in four quadrant CELLview culture dishes (VWR, Radnor, PA.) with RPMI complete medium, allowed to adhere for 30 hours and then treated with C-PAC [50 μg/ml] for 24 hours. Growth medium was removed and cells were stained with Monodansylcadaverine (MDC) (Sigma-Aldrich #30432) for 30 min at 37°C to label acidic autophagic vacuoles which are part of the lysosomal compartment. Confocal microscopy was utilized to visualize the MDC fluorescence (335/525 nm, excitation/emission).

### Xenograft EAC tumor model

All experimental procedures were conducted in accordance with a protocol approved by the Institutional Laboratory Animal Care and Use Committee of the University of Miami. Twelve 5-6 week old male NU/NU athymic mice were purchased from Charles Rivers Laboratories, Wilmington, MA. Mice were fed irradiated synthetic AIN93G diet and provided water *ad libitum*. OE19 cells were suspended in RPMI medium and matrigel and subcutaneously implanted at a concentration of 1.25E6 cells in each flank; cells grew for 5 days reaching a mean tumor volume of 150 mm^3^ prior to mice being randomized to vehicle or C-PAC treatment groups. C-PAC was delivered by oral gavage (100 μl) at a concentration of 250 μg/mouse, 6 days a week. Tumors were measured every 2-3 days throughout the experiment and mean tumor volume calculated using the following formula: *π/6 x L x W x H* (*L*, length; *W,* width; *H,* height). Increasing tumor size in the vehicle treated group required termination of the experiment on day 19. OE33 and JHAD1 cells did not generate tumors in this animal model, even when higher concentrations of cells were injected supporting that OE19 cells are phenotypically more aggressive.

### Statistical analysis

Two-tailed unpaired Student's *t*-test or ANOVA were employed to test for statistically significant differences between treatment groups, followed by Tukey's post-hoc test. GraphPad Prism version 6.0 was utilized with *P* < 0.05 considered statistically significant.

## SUPPLEMENTARY MATERIAL FIGURES


