# Supplementary material for: Cranberry proanthocyanidins inhibit esophageal adenocarcinoma in vitro and in vivo through pleiotropic cell death induction and PI3K/AKT/mTOR inactivation
Source: Oncotarget. 2015 Sep 10;6(32):33438–55. doi: 10.18632/oncotarget.5586 (PMC4741777; doi:10.18632/oncotarget.5586)
Supplement: Supplementary file 1 [file oncotarget-06-33438-s001.pdf]

Cranberry proanthocyanidins inhibit esophageal adenocarcinoma *in vitro* and *in vivo* through pleiotropic cell death induction and PI3K/AKT/mTOR inactivation

Supplementary Material

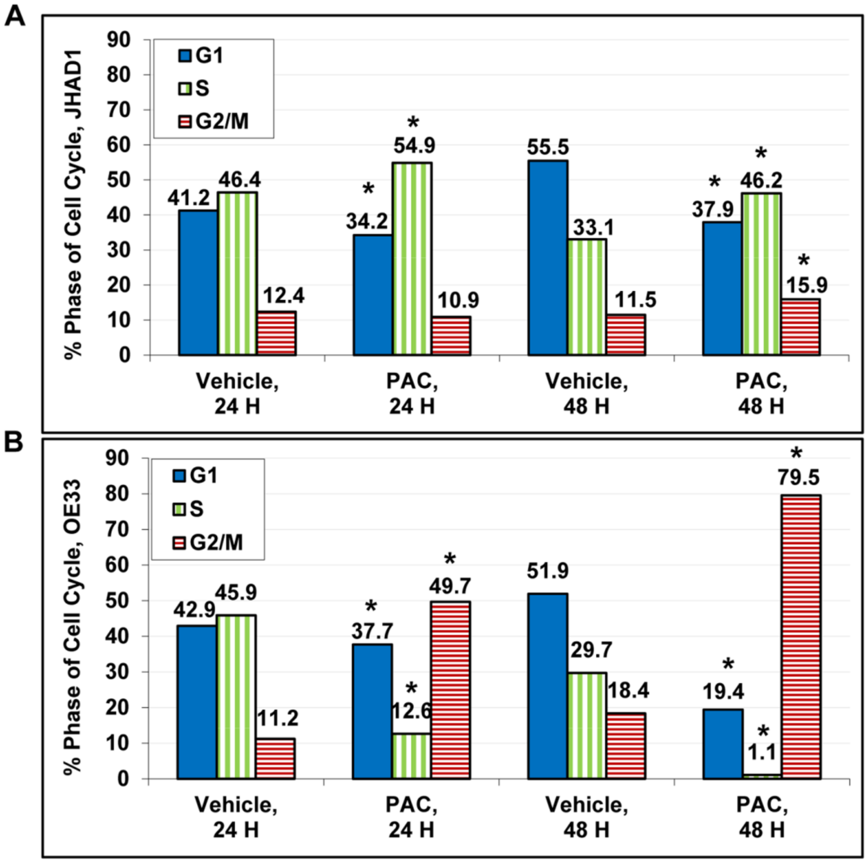

Supplemental Figure 1S. Effects of C-PAC [50 µg/ml] on phase of cell cycle distribution and cell death induction· A) Cell cycle distribution in JHAD1 cells and B) Cell cycle distribution in OE33 cells following 24 and 48 hours of C-PAC treatment. C) C-PAC treatment significantly altered phase of cell cycle distribution. The upper left and upper right quadrants of each treatment panel represent early and late S-phase, respectively. C-PAC treatment resulted in a significant reduction in S-phase fraction, a significant reduction in G_1 cells and a significant increase in cells stacking up at G_2-M. D) Cellular apoptosis and necrosis inducing effects of C-PAC in OE33 cells. $^*P<0.05$ indicates a significant difference between C-PAC and vehicle treated cells, two-tailed Students *t*-test. C-PAC treatment of OE33 cells induced significant apoptosis and necrosis at 24 hours with continued necrosis at 48 hours.

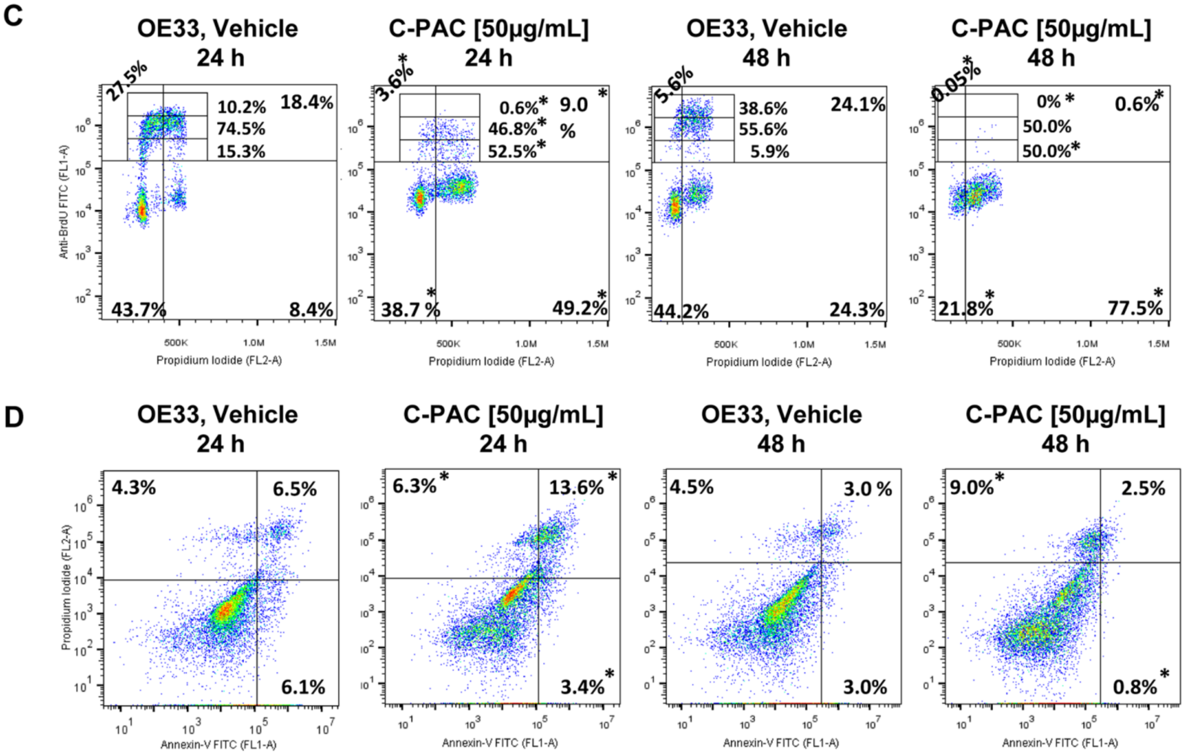

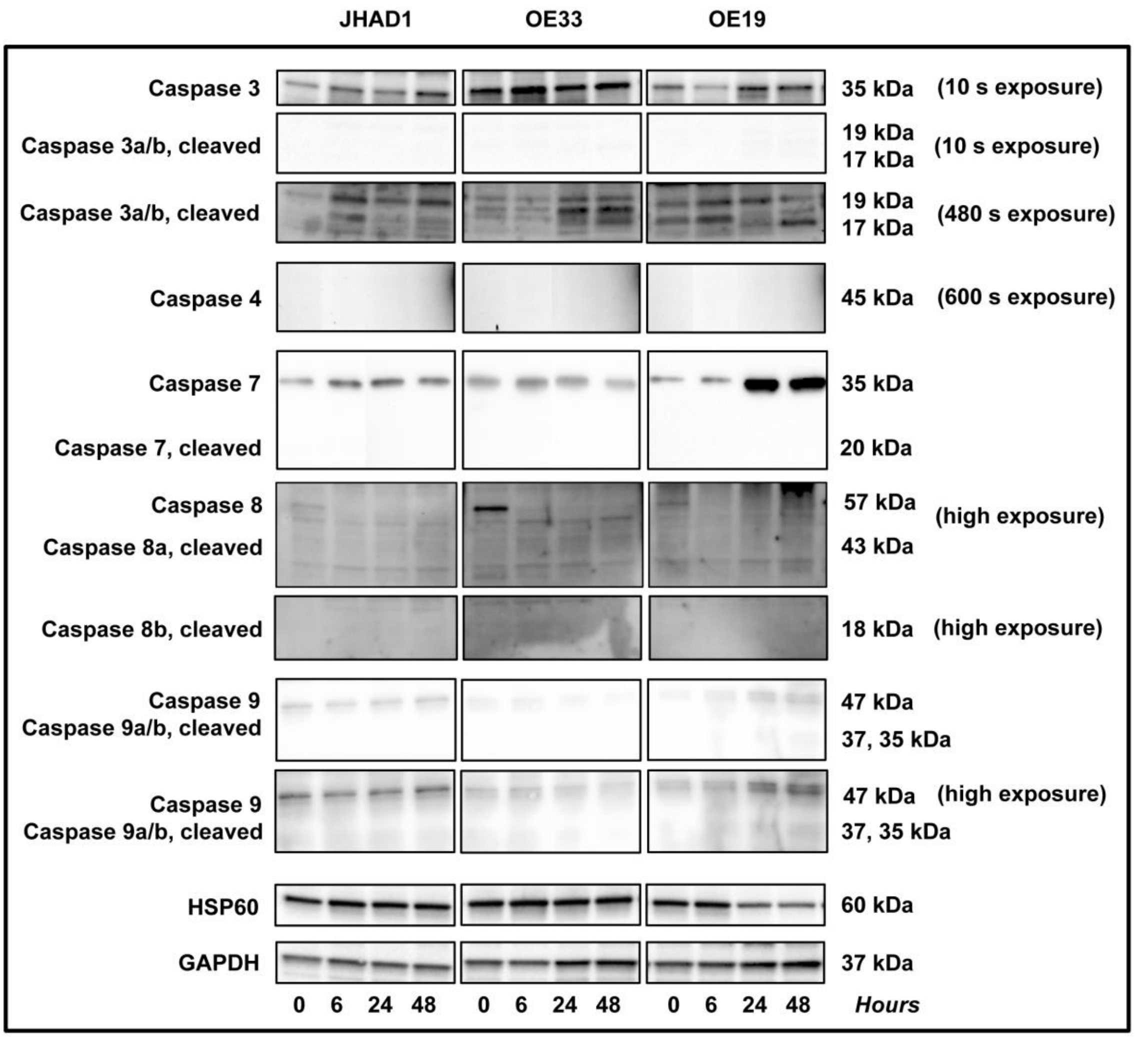

Supplemental Figure 2S. Effects of C-PAC [50 μg/ml] on expression of caspases in EAC cells. JHAD1, OE33 and OE19 cells were treated with of C-PAC [50 μg/ml] and lysates harvested at pretreatment or baseline and at 6, 24 and 48 hours following treatment. Immunoblot was performed using commercially available antibodies to the proteins of interest as detailed in the materials and methods section. Expression values were normalized to the appropriate loading control, GAPDH or HSP60.
